# Systemic and sustained thioredoxin analogue prevents acute kidney injury and its-associated distant organ damage in renal ischemia reperfusion injury mice

**DOI:** 10.1038/s41598-020-75025-5

**Published:** 2020-11-26

**Authors:** Kento Nishida, Hiroshi Watanabe, Masako Miyahisa, Yuto Hiramoto, Hiroto Nosaki, Rui Fujimura, Hitoshi Maeda, Masaki Otagiri, Toru Maruyama

**Affiliations:** 1 Department of Biopharmaceutics, Graduate School of Pharmaceutical Sciences, Kumamoto University, 5-1, Oe-honmachi, Chuo-ku, Kumamoto, 862-0973 Japan; 2 Center for Clinical Pharmaceutical Sciences, School of Pharmacy, Kumamoto University, 5-1, Oe-honmachi, Chuo-ku, Kumamoto, 862-0973 Japan; 3 Faculty of Pharmaceutical Sciences, Sojo University, 4-22-1 Ikeda, Kumamoto, 860-0082 Japan

**Keywords:** Drug delivery, Acute kidney injury, Respiratory distress syndrome

## Abstract

The mortality of patients with acute kidney injury (AKI) remains high due to AKI associated-lung injury. An effective strategy for preventing both AKI and AKI-associated lung injury is urgently needed. Thioredoxin-1 (Trx) is a redox-active protein that possesses anti-oxidative, anti-apoptotic and anti-inflammatory properties including modulation of macrophage migration inhibitory factor (MIF), but its short half-life limits its clinical application. Therefore, we examined the preventive effect of a long-acting Trx, which is a fusion protein of albumin and Trx (HSA-Trx), against AKI and AKI-associated lung injury. Recombinant HSA-Trx was expressed using a *Pichia* expression system. AKI-induced lung injury mice were generated by bilateral renal ischemia reperfusion injury (IRI). HSA-Trx administration attenuated renal IRI and its-associated lung injury. Both renal and pulmonary oxidative stress were suppressed by HSA-Trx. Moreover, HSA-Trx inhibited elevations of plasma IL-6 and TNF-α level, and suppressed IL-6–CXCL1/2-mediated neutrophil infiltration into lung and TNF-α-mediated pulmonary apoptosis. Additionally, HSA-Trx suppressed renal IRI-induced MIF expression in kidney and lung. Administration of HSA-Trx resulted in a significant increase in the survival rate of renal IRI mice. Collectively, HSA-Trx could have therapeutic utility in preventing both AKI and AKI-associated lung injury as a consequence of its systemic and sustained multiple biological action.

## Introduction

Acute kidney injury (AKI) is characterized by abrupt impairment of renal function within a short period of time and is widely recognized as a complication in patients after surgery. The incidence of cardiac surgery-associated AKI is particularly high at 20–40%, resulting in increased mortality of patients undergoing this procedure^1,2^. The mortality of severe AKI cases, defined as stage 3 by kidney disease improving global outcomes (KDIGO), is 62% for those requiring dialysis and 41% for those that do not^3^. The reason for the high level of mortality could be the development of distant organ injury associated with AKI, which is characterized by extra-renal organ injury, such as lung and liver. Unfortunately, the prognosis of AKI patients with lung injury is poor. It was reported that the mortality odds ratio of AKI-associated lung injury is 10.3 compared with 1.7 for AKI-associated non-respiratory organ injury^4^. Thus, a new therapeutic approach for preventing not only cardiac surgery-associated AKI but also AKI-associated extra-renal organ injury, such as lung injury, is urgently needed.

In terms of development of AKI-associated lung injury, pulmonary endothelial and epithelial cells damage after AKI results in pulmonary hyper-permeability, leading to pulmonary alveolar edema and ventilatory failure^5,6^. Recent studies suggest that numerous complex interactions are involved in pulmonary cell damage, including neutrophil infiltration, production of reactive oxygen species (ROS), apoptosis and cytokine production. Three cascades of events leading from AKI to lung injury have been reported: (1) IL-6-mediated neutrophil infiltration into lung^7,8^, (2) TNF-α-mediated pulmonary apoptosis^9–11^, and (3) an increase in plasma cytokine levels derived from extra-renal organs such as liver^12–14^. Firstly, pro-inflammatory cytokines, such as IL-6 and TNF-α, are produced by injured kidney that then reach the lung via the bloodstream. In lung, IL-6 binds to IL-6 receptor, resulting in production of chemokines CXCL1 and CXCL2, which promote neutrophil infiltration into lung. The infiltrated neutrophils produce ROS, such as superoxide and hypochlorous acid via NADPH oxidase and myeloperoxidase (MPO), respectively, leading to pulmonary cell damage. In addition, TNF-α binds to TNF receptor on pulmonary endothelial cells, resulting in apoptosis. After AKI, extra-renal tissue such as liver also produces inflammatory cytokines, which contribute to their elevation in the plasma thereby further accelerating lung injury via cytokine-mediated cascades. Based on this evidence in which oxidative stress, apoptosis and inflammation are strongly associated with the development of AKI and its complications, a prophylactic agent is needed to exert systemic and sustained anti-oxidative, anti-apoptotic and anti-inflammatory actions.

Thioredoxin-1 (Trx) is a redox-active low-molecular-weight protein that has a suppressive effect on oxidative stress, apoptosis and inflammation including modulation of macrophage migration inhibitory factor (MIF). Secreted MIF in response to several stress factors activate immune cells such as macrophages, which produce pro-inflammatory cytokines^15^. Thus, MIF has recently been recognized as an aggravating factor for inflammatory diseases, including acute lung injury. Although Trx is a promising candidate for the treatment of oxidative stress and inflammation-associated diseases^16^, its plasma half-life is extremely short (1 or 2 h in mouse and rat, respectively), which limits its clinical application. We recently engineered a genetic fusion protein consisting of human serum albumin (HSA) and Trx (HSA-Trx) and expressed it in a *Pichia* system^17^. In terms of its pharmacokinetic properties, the HSA-Trx fusion protein is similar to HSA, while the plasma half-life of HSA-Trx was increased by approximately tenfold compared with that of Trx^17–19^. Interestingly, compared with Trx, the lung distribution of HSA-Trx is fivefold higher^17^. HSA-Trx also exhibited a greater distribution in the kidney and liver compared to other organs^17^. To date, we have demonstrated that HSA-Trx has therapeutic benefit against oxidative stress-associated diseases, including kidney^20–22^, lung^19,23^ and liver disease^24^. The therapeutic utility of HSA-Trx is based on its anti-oxidative, anti-inflammatory properties and anti-apoptotic actions. Thus, due of its systemic and sustained multiple biological effects, we reasoned that HSA-Trx may prevent both AKI and AKI-associated lung injury.

## Results

### Mouse model of AKI-associated lung injury

The mouse model of AKI-associated lung injury was induced by renal IRI where both renal pedicles were clamped for 60 min (Supplemental Fig. 1A). These mice exhibited elevations in BUN and Scr in a time-dependent manner for 36 h after renal IR (Supplemental Fig. 1B). In addition, an increased protein concentration in BALF, which is a marker of pulmonary hyper-permeability, was observed at 24 and 36 h after renal IR (Supplemental Fig. 1C), as reported previously^9,25^. These data demonstrated the suitability of this renal IRI-associated lung injury model for the following study to evaluate the potential preventive effect of HSA-Trx.

### Effect of HSA-Trx on renal IR-induced renal dysfunction and histological alterations

**Figure 1:**
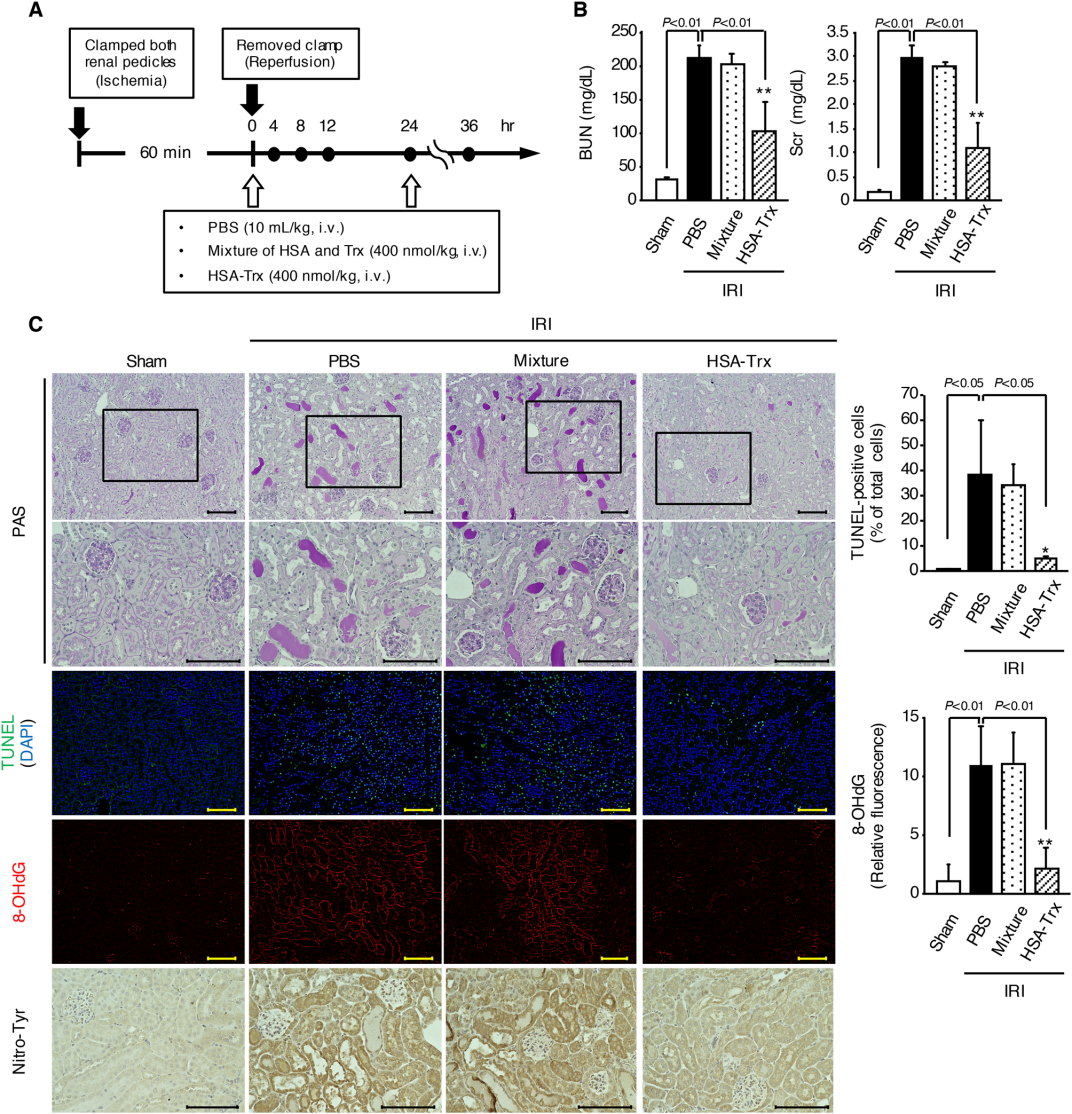
Renoprotective effect of HSA-Trx against renal ischemia reperfusion-induced AKI via suppression of apoptosis and oxidative stress. (**A**) Dosing schedule of HSA-Trx on renal ischemia reperfusion (IR)-induced AKI. The mixture of HSA plus Trx or HSA-Trx alone was administered *i.v.* at a dose of 400 nmol/kg, immediately and 24 h after renal IR. An equivalent amount of phosphate-buffered saline (PBS) was administered to the sham operation group and the renal-IR group. (**B**) Blood urea nitrogen (BUN) and serum creatinine (Scr) levels were measured at 36 h after renal IR. (**C**) Representative photomicrographs of renal histological features (PAS staining), renal apoptosis (TUNEL staining), immunostaining of renal 8-hydroxy-2′-deoxyguanosine (8-OHdG), which is an oxidative stress marker of nucleic acid, and renal nitrotyrosine (Nitro-Tyr), which is an oxidative stress marker of amino acids, are shown at 36 h after renal IR. Lower panels of PAS are an enlarged image of the upper panel. TUNEL (green)-stained kidney sections were also treated with DAPI (blue). Original magnifications: × 200 (upper panels of PAS, TUNEL and 8-OHdG); × 400 (lower panels of PAS and Nitro-Tyr). Scale bars represent 100 μm. Percentage (%) of TUNEL and DAPI double-positive cells relative to total cells (DAPI-positive cells) are indicated. Image analysis was performed to quantify the extent and intensity of 8-OHdG staining. Data are expressed as means ± SD (n = 5–6). *P < 0.05, **P < 0.01 compared with renal IR-mice administered with the 1:1 mixture of HSA and Trx.

The experimental protocol is summarized in Fig. 1A. Our previous study clearly indicated the renoprotective effect of HSA-Trx against glycerol-induced AKI mice and showed that HSA-Trx exerted a therapeutic effect in a dose-dependent manner (100–400 nmol/kg)^22^. Based on these findings, the dose of HSA-Trx used in this study was set at 400 nmol/kg. Compared to the PBS-treated group, intravenous injection of HSA-Trx immediately and 24 h after renal IR significantly attenuated the elevation in BUN and Scr levels 36 h after renal IR (Fig. 1B). PAS staining was also performed to evaluate histological alterations of the kidneys (Fig. 1C). At 36 h after renal IR the PBS-treated group showed tubular cell damage and cast formation compared with the sham group, while such histological alterations were greatly suppressed by HSA-Trx. These histological changes are consistent with the altered BUN and Scr as shown in Fig. 1B. In contrast to HSA-Trx treatment, administration of a 1:1 mixture of HSA and Trx did not affect renal dysfunction (Fig. 1B) or histological alterations (Fig. 1C) caused by renal IR. These data indicate that HSA-Trx attenuated kidney injury caused by renal IR.

### Effect of HSA-Trx on renal tubular apoptosis and renal oxidative stress caused by renal IR

To evaluate renal tubular apoptosis, TUNEL staining was performed (Fig. 1C). Kidneys from renal IR mice administered with PBS or the mixture of HSA and Trx exhibited an increased number of TUNEL-positive tubular epithelial cells. By contrast, the administration of HSA-Trx markedly decreased the number of TUNEL-positive cells. It has been demonstrated that the production of ROS and redox imbalance are critical in the development of renal IR-induced kidney injury^26,27^. Therefore, immunostaining of kidneys from renal IR mice with 8-OHdG or Nitro-Tyr was performed to examine whether HSA-Trx caused a decrease in renal IR-induced ROS (Fig. 1C). Compared with sham mice, 8-OHdG and Nitro-Tyr-positive cells were markedly increased in the renal tubules of renal IR-mice administered with PBS or the mixture of HSA plus Trx. In contrast, HSA-Trx treatment clearly inhibited the increased number of 8-OHdG and Nitro-Tyr-positive cells. These results suggest that HSA-Trx suppressed renal tubular apoptosis and oxidative stress caused by renal IR.

### Effect of HSA-Trx on pulmonary histological changes and endothelial hyper-permeability induced by renal IRI

**Figure 2:**
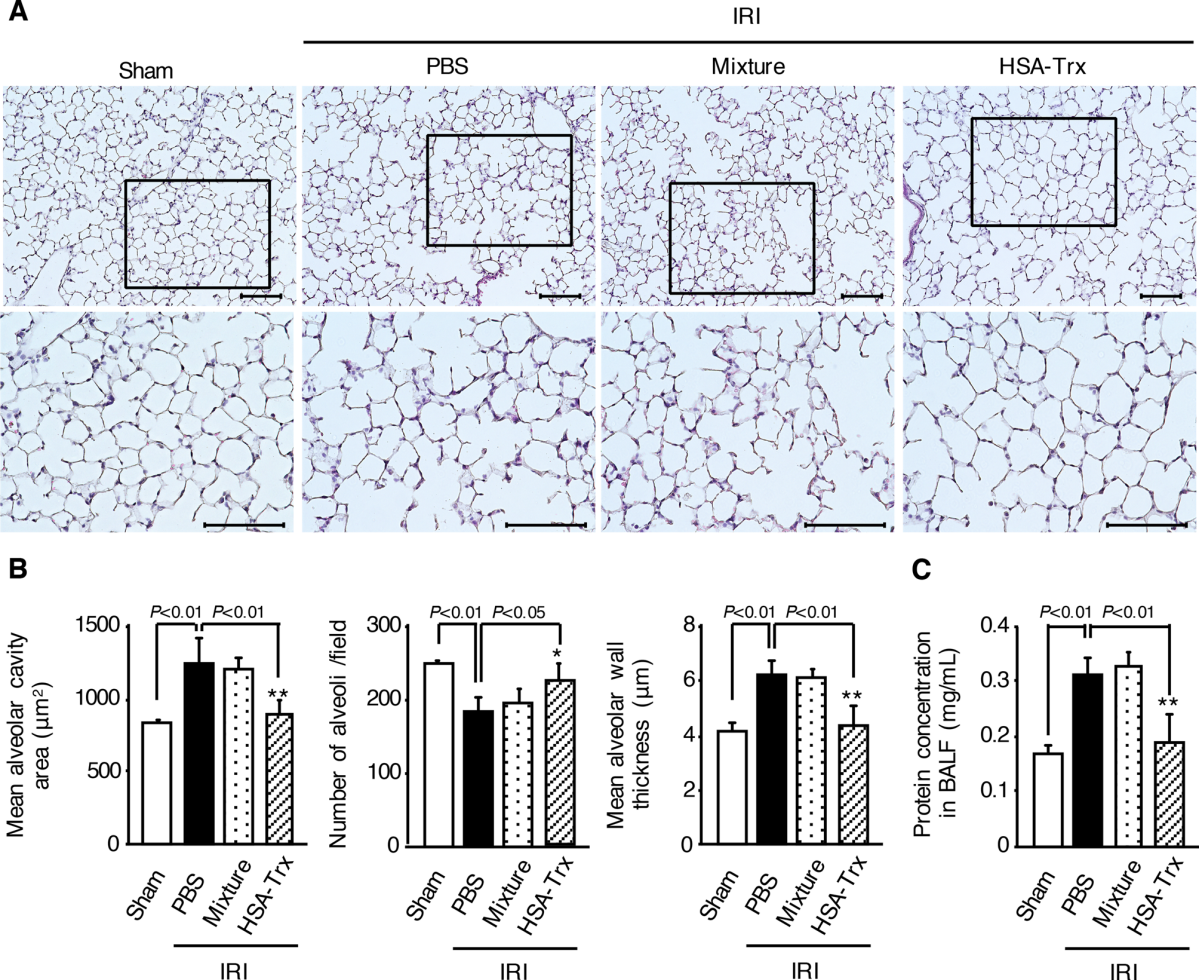
Protective effect of HSA-Trx against lung injury in renal IR-mice. (**A**) Representative micrographs of HE-stained lung tissues and (**B**) a semi-quantitative scoring analysis of alveolar cavity area, the number of alveoli, and alveolar wall thickness at 36 h after renal IR in sham mice and renal IR-mice treated with PBS, a mixture of HSA and Trx, or HSA-Trx alone. Lower panels of HE show an enlarged image of the upper panel. Original magnifications: × 200 (upper panels); × 400 (lower panels). Scale bars represent 100 μm. Four high-magnification (× 200) fields in a lung section were randomly selected for the semi-quantitative analysis of morphological changes. (**C**) Protein concentration in BALF, which is increased by pulmonary hyper-permeability, was determined at 36 h after renal IR. Data are expressed as the means ± SD (n = 5–6). *P < 0.05, **P < 0.01 compared with renal IR-mice administered with the 1:1 mixture of HSA plus Trx.

To evaluate lung injury induced by renal IRI, HE staining (Fig. 2A,B) and measurements of protein concentration in BALF (Fig. 2C) were performed. Compared with the sham group, pulmonary histological alterations such as an increase of alveolar cavity area and decrease in the number of alveoli, which represents alveolar structural disorder, were observed in the PBS group at 36 h after renal IR. Moreover, the PBS group showed an increase of alveolar wall thickness and protein level in BALF, which represents pulmonary edema and endothelial hyper-permeability, respectively. However, these histological features and the change of protein level in BALF were largely inhibited by administration of HSA-Trx. These results indicate that HSA-Trx prevented renal IRI-induced lung injury. In contrast to the HSA-Trx treatment, the mixture of HSA plus Trx did not affect renal IRI-associated histological alterations and endothelial hyper-permeability in lung. Based on the results shown in Figs. 1 and 2, the protective effect of HSA-Trx against renal and lung injury could be due to sustained Trx activity by fusion with HSA.

### Effect of HSA-Trx on pulmonary redox imbalance induced by renal IRI

**Figure 3:**
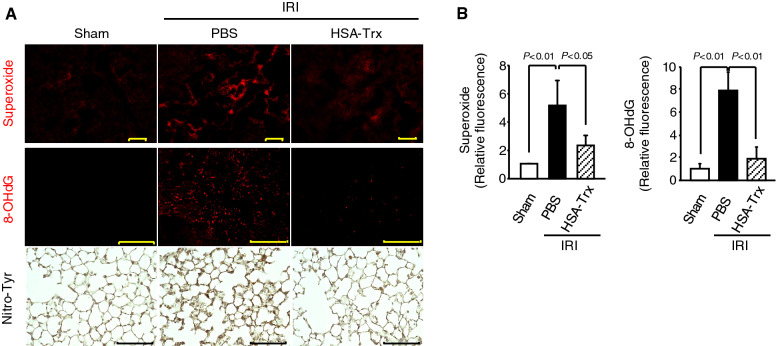
The effect of HSA-Trx on pulmonary redox imbalance in renal IR-mice. (**A**) Representative micrographs of pulmonary superoxide (upper panels), detected by DHE staining, 8-OHdG (middle panels) and Nitro-Tyr (lower panels) are presented. DHE staining, or immunostaining of 8-OHdG and Nitro-Tyr were performed 8 h or 36 h after renal IR with or without HSA-Trx treatment. Original magnifications: × 200 (superoxide); × 400 (8-OHdG and Nitro-Tyr). Scale bars represent 100 μm. (**B**) Image analysis was performed to quantify the extent and intensity of DHE and 8-OHdG staining. Data are expressed as means ± SD (n = 4).

The production of ROS, such as superoxide derived from activated neutrophils, is critical in the development of AKI-associated lung injury^28,29^. Therefore, lung sections at 8 h after renal IR were examined for superoxide production (DHE staining) to assess whether HSA-Trx caused a decrease in renal IR-induced pulmonary ROS (Fig. 3A upper panels,B). In addition, lung sections at 36 h after renal IR were also immunostained for 8-OHdG (Fig. 3A middle panels,B) and Nitro-Tyr (Fig. 3A lower panels). Renal IR-mice administered with PBS exhibited an increased number of superoxide, 8-OHdG and Nitro-Tyr-positive cells in lung compared to the sham mice. In contrast to the PBS treatment, HSA-Trx clearly suppressed the increased number of superoxide, 8-OHdG and Nitro-Tyr-positive cells. These results suggest that HSA-Trx suppressed pulmonary oxidative stress in renal IRI-associated lung injury mice.

### Plasma IL-6 and neutrophil in BALF induced by renal IRI

IL-6 produced from injured kidney reaches the lungs via the bloodstream, where it induces the expression of chemokines CXCL1 and CXCL2^7,8^. These chemokines promote neutrophil infiltration and ROS production from neutrophils, resulting in lung injury (e.g. pulmonary hyper-permeability). In fact, the mouse model of AKI-associated lung injury used in this study exhibited elevations in plasma IL-6 level, reaching a peak 4 h after renal IR (Supplemental Fig. 2A). In addition, an increase of neutrophils in BALF was observed after renal IR, which peaked 8 h after renal IR (Supplemental Fig. 2B).

### Effect of HSA-Trx on IL-6-mediated neutrophil infiltration into lung by renal IRI

**Figure 4:**
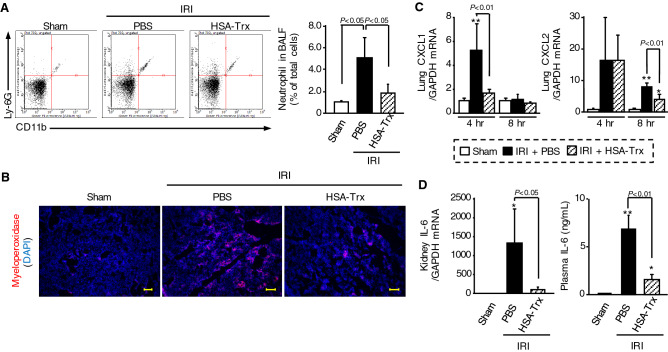
Effect of HSA-Trx on neutrophil infiltration into lung in renal IR-mice. (**A**) Representative FACS dot plots showing the expression of CD11b and Ly-6G in BALF cells from mice at 8 h after sham operation or renal IR with or without HSA-Trx treatment. Percentage (%) of neutrophil (CD11b and Ly-6G double-positive cells) relative to total cells in BALF are indicated. (**B**) Immunostaining of pulmonary myeloperoxidase (MPO), which is a marker of neutrophils, at 8 h after renal IR. MPO (red)-stained lung sections were also treated with DAPI (blue). Original magnifications: × 100. Scale bars represent 100 μm. (**C**) CXCL1 and CXCL2 mRNA expression in lung at 4 and 8 h after renal IR were determined by real-time RT-PCR. (**D**) Renal mRNA and plasma level of IL-6 at 4 h after renal IR were determined by real-time RT-PCR and ELISA, respectively. Data are expressed as means ± SD (n = 4–6). *P < 0.05, **P < 0.01 compared with sham at each time point.

We investigated whether HSA-Trx attenuates renal IRI-induced lung injury via suppressing IL-6–CXCL1/2-mediated neutrophil infiltration into lung. Neutrophils infiltration in BALF was measured as Ly-6G and CD11b double-positive cells by flowcytometry. At 8 h after renal IR, HSA-Trx treatment suppressed the increase in neutrophils in BALF (Fig. 4A). Moreover, MPO (a marker of neutrophil-positive cells in lung) also decreased after HSA-Trx treatment (Fig. 4B). Renal IR-mice treated with PBS showed an increase in CXCL1 and CXCL2 mRNA expression levels compared to the sham mice at the 4 h time point (Fig. 4C). In contrast, the HSA-Trx treatment suppressed CXCL1 or CXCL2 expression at 4 h or 8 h after renal IR, respectively, compared with the PBS treatment. At 4 h after renal IR, mRNA expression of IL-6 in kidney was also increased as well as the level of plasma IL-6 (Fig. 4D). However, renal mRNA expression and plasma levels of IL-6 protein were suppressed by HSA-Trx administration. These data suggest that HSA-Trx prevents renal IRI-induced lung injury by suppressing IL-6–CXCL1/2-mediated neutrophil infiltration into lung.

### Effect of HSA-Trx on TNF-α-mediated pulmonary apoptosis induced by renal IRI

**Figure 5:**
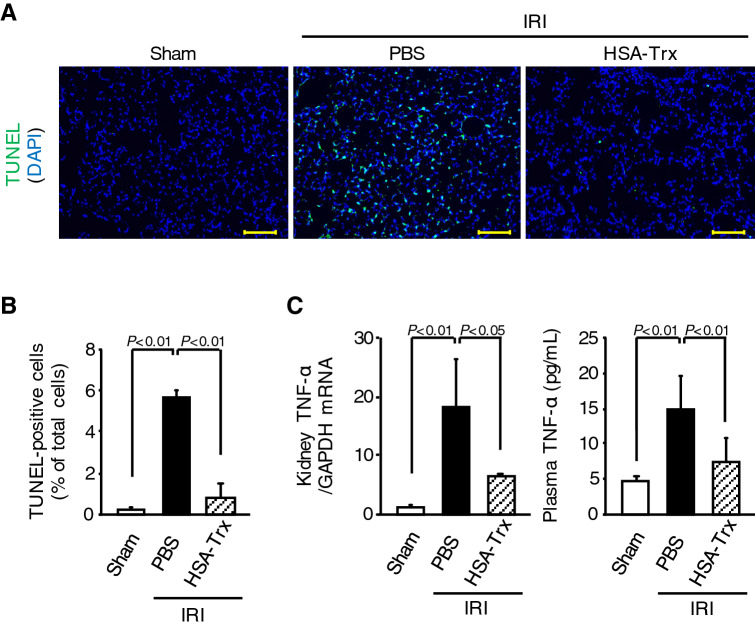
Effect of HSA-Trx on TNF-α-mediated pulmonary apoptosis in renal IR-mice. (**A**) Representative micrographs of TUNEL (green) and DAPI (blue)-double stained lung tissue are shown 36 h after renal IR with or without HSA-Trx treatment. Original magnifications: × 200. Scale bars represent 100 μm. (**B**) Percentage (%) of TUNEL and DAPI double-positive cells relative to total cells (DAPI-positive cells) are indicated. (**C**) mRNA expression of TNF-α in kidney 4 h after renal IR and plasma TNF-α level 36 h after renal IR were determined by real-time RT-PCR and ELISA, respectively. Data are expressed as means ± SD (n = 4–6).

Like IL-6, TNF-α is also produced from injured kidney, which then induces pulmonary apoptosis after reaching the lung^9,10^. Indeed, the mouse model used in this study exhibited elevations in plasma TNF-α level at 36 h after IR (Supplemental Fig. 2C). From these finding, we investigated whether HSA-Trx attenuates renal IRI-induced lung injury via suppressing TNF-α-mediated pulmonary apoptosis. Lung from the renal IR-mice treated with PBS showed a marked increase in the number of TUNEL-positive cells (Fig. 5A,B). In contrast to the PBS group, the number of TUNEL-positive cells in the HSA-Trx group was significantly decreased. In addition, HSA-Trx treatment significantly suppressed the increase of renal mRNA expression or plasma protein levels of TNF-α at 4 or 36 h after renal IR, respectively (Fig. 5C). These results suggest that HSA-Trx inhibited AKI-induced lung injury by suppressing TNF-α-mediated pulmonary apoptosis.

### Effect of HSA-Trx on liver injury caused by renal IRI

**Figure 6:**
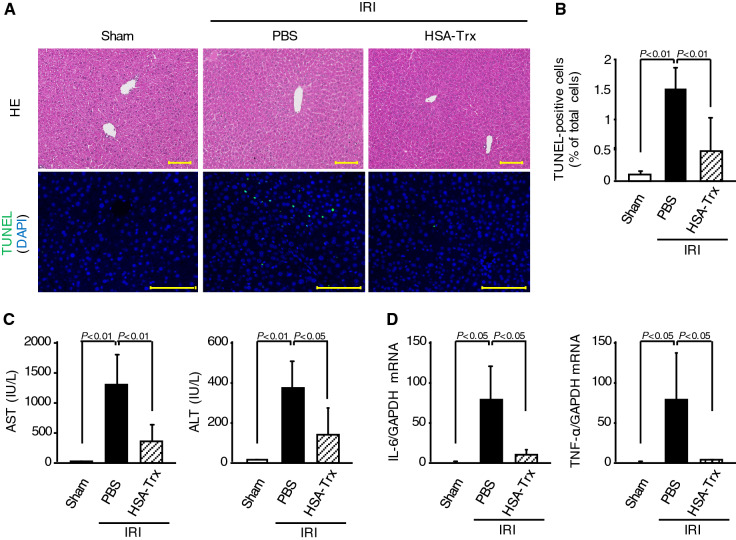
Effect of HSA-Trx on liver injury and cytokine expression in the liver of renal IR-mice. (**A**) Representative micrographs of HE-stained and TUNEL (green) and DAPI (blue)-double stained liver tissue are shown 36 h after renal IR with or without HSA-Trx. Original magnifications: × 200 (HE); × 400 (TUNEL). Scale bars represent 100 μm. (**B**) Percentage (%) of TUNEL and DAPI double-positive cells relative to total cells (DAPI-positive cells) are indicated. (**C**) Aspartate aminotransferase (AST) and alanine aminotransferase (ALT) levels were measured 36 h after renal IR. (**D**) mRNA expressions of IL-6 and TNF-α in liver 4 h after renal IR were determined by real-time RT-PCR. Data are expressed as means ± SD (n = 4–6).

AKI causes not only lung injury but also other organ injury such as liver injury^12–14^. Andrés-Hernando et al. reported that the injured liver after AKI also released inflammatory cytokines such as IL-6, TNF-α, which could contribute to kidney-lung crosstalk^13^. Thus, we investigated whether HSA-Trx attenuates liver injury. Liver from renal IR-mice treated with PBS or HSA-Trx did not show clear histological alterations compared with sham. Although a slight increase in the number of TUNEL-positive cells was observed at 36 h after renal IR (Fig. 6A,B), the HSA-Trx treatment decreased the number of TUNEL-positive cells to the same level as the sham group. Moreover, HSA-Trx significantly attenuated the increase of AST and ALT levels at 36 h after renal IR in comparison with the PBS treatment (Fig. 6C). Similarly, an increase of mRNA expression of IL-6 and TNF-α in liver was significantly inhibited by HSA-Trx treatment (Fig. 6D). These results suggest that HSA-Trx prevented renal IRI-induced liver injury.

### Effect of HSA-Trx on renal IR-induced MIF expression

**Figure 7:**
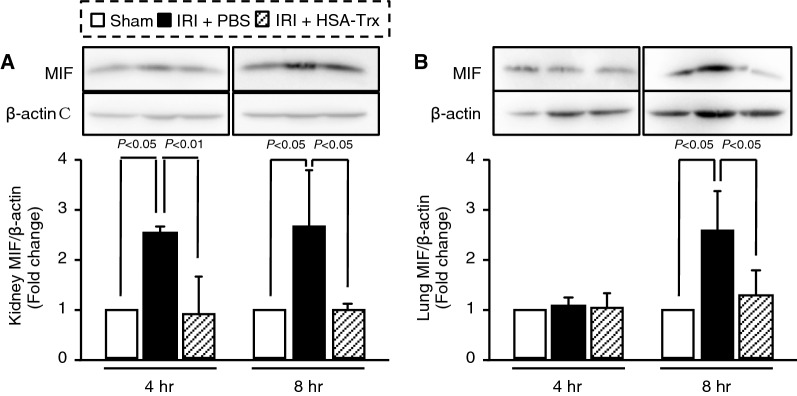
Effect of HSA-Trx on the expression of MIF in renal IRI-induced lung injury mice. MIF expression in (**A**) kidney and (**B**) lung 4 and 8 h after renal IR was assessed by Western blotting. The intensity of each band was quantified using ImageJ software and normalized against β-actin expression. Data are expressed as means ± SD (n = 4).

Our previous study showed MIF is a cytokine that acts as an exacerbation factor in the development of inflammation-related disease including AKI^22^ and lung injury^19^. Hence, we also evaluated the effect of HSA-Trx on MIF expression in kidney and lung at 4 and 8 h after renal IR. As shown in Fig. 7A, MIF expression levels in the kidney of renal IR-mice was significantly increased at least from 4 h after renal IR in comparison to sham mice, whereas HSA-Trx treatment suppressed the increase of MIF expression. In the lung, MIF expression levels at 4 h after renal IR did not show significant differences compared with sham mice, but at 8 h after renal IR, the expression was significantly increased (Fig. 7B). By contrast, the HSA-Trx-treated group showed a suppression of MIF expression in lung at 8 h after IR. These results suggest that HSA-Trx exerts an anti-inflammatory activity in part at least via suppressing MIF expression on renal IRI-associated lung injury.

### Effect of HSA-Trx on the survival of renal IRI-associated lung injury mice

**Figure 8:**
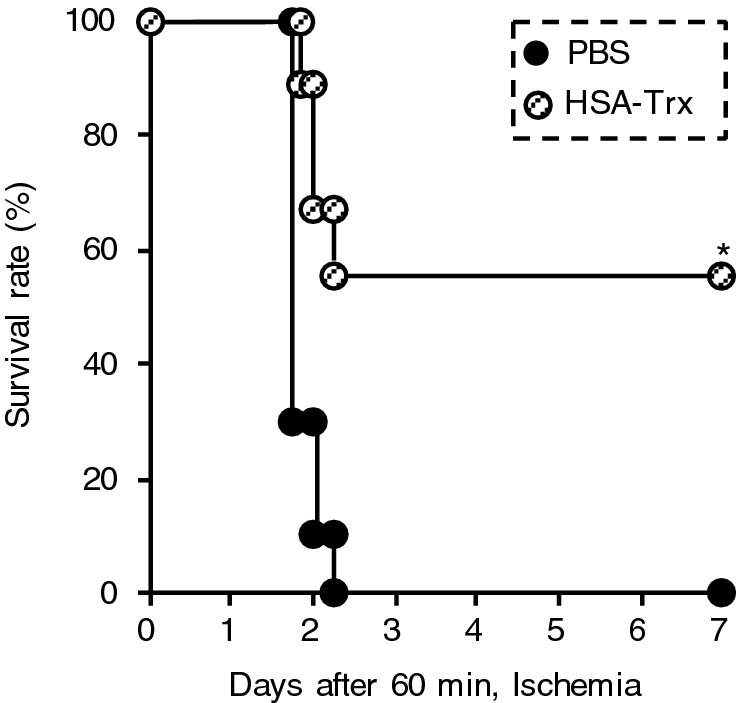
Effect of HSA-Trx on the survival of renal IRI-associated lung injury mice. PBS or HSA-Trx were intravenously administered immediately and 24 h after renal IR. The number of mice (n) in each group were 9. *P < 0.05 compared with renal IR-mice administered with PBS.

Finally, we evaluated the effect of HSA-Trx on the survival rate of renal IRI-associated lung injury mice (Fig. 8). All of the renal IR-treated mice that were administered PBS died within three days of IR. By contrast, HSA-Trx treatment increased the survival rate, with 55% of the mice still alive at seven days.

## Discussion

The incidence and the mortality of patients with AKI after cardiac surgery remains high. Meersch et al. recently reported that implementation of the KDIGO guidelines significantly reduced the occurrence and severity of AKI after cardiac surgery in patients at high risk of AKI onset^30^. Nonetheless, the occurrence of AKI in the intervention group is still high at 55.1%. Moreover, this intervention had no effect on mortality compared with the control group, indicating that the intervention against cardiac surgery-associated AKI should be further investigated. Distant organ complications after AKI greatly contribute to an increase in mortality of AKI patients. Respiratory complication is an important consideration in AKI patients, because previous reports showed that the onset of lung injury in AKI patients gave the worst prognosis among all AKI-associated distant organ injury^4^ and a negative spiral of kidney-lung crosstalk. Thus, AKI induces pulmonary injury, and in turn, AKI-induced lung injury accelerates kidney injury^31^. Lee et al. has proposed therapeutic strategies that target just a single molecule or single pathway are less likely to succeed in reducing the AKI-induced distant organ dysfunctions because of their complex interactions during AKI^6^. From this perspective, systemic and sustained Trx analogue has a potential therapeutic benefit due to its multiple biological actions including anti-oxidative activity, anti-inflammatory and anti-apoptotic. Indeed, the present study showed that HSA-Trx effectively ameliorated both AKI and AKI-associated distal organ damage in bilateral renal IRI model mice, which mimics cardiac surgery-associated AKI.

It has been demonstrated that various inflammatory changes occur in renal IRI-induced lung injury. As shown in Figs. 4, 5, 6, pro-inflammatory cytokines, including IL-6 and TNF-α, were increased after renal IRI, while HSA-Trx treatment markedly suppressed expression of these cytokines in plasma, kidney and lung. IL-6 is considered to act as a mediator in lung injury via the induction of chemokines such as CXCL1 and CXCL2, which subsequently coordinate the infiltration of neutrophils into the lung^7,8^. TNF-α is also thought to contribute to the development of AKI-associated lung injury via TNF-α/TNF receptor signaling on pulmonary endothelial cells followed by the induction of caspase-dependent pulmonary apoptosis^9–11^. With regard to a therapeutic approach focusing on IL-6 and TNF-α, Klein et al. reported that ischemic AKI and bilateral nephrectomy-induced neutrophil infiltration into lung can be inhibited by administration of an anti-IL-6 antibody^7^. White et al. also reported that Etanercept, a TNF inhibitor, ameliorated ischemic AKI-induced pulmonary apoptosis^10^. These previous data support the findings from our study, which suggests that HSA-Trx treatment prevents AKI-induced lung injury via suppression of IL-6 and TNF-α expression.

In this study, it appears that improvement in the inflammatory condition in three tissues by HSA-Trx treatment could arise, in part, from inhibition of MIF expression because Trx has been reported to exhibit its anti-inflammatory activities via modulating the expression of MIF^32,33^. Previously, Tamaki et al. reported that the expression and secretion of MIF in human monocyte cells is suppressed by exogenous Trx treatment, implying MIF and Trx counteract each other during the inflammatory response^33^. Under oxidative stress and inflammation-associated pathologic conditions, expression and production of MIF is up-regulated^34^. Moreover, a previous study showed that pulmonary MIF levels are increased and then contribute to pathogenesis via the activation of pulmonary macrophages in a lung disorder model such as acute respiratory distress syndrome^35^, acute pancreatitis-induced lung injury^36^ and intestinal IRI-induced lung injury^37^. In such cases, MIF facilitates expression of IL-6, TNF-α, etc., as well as ROS production including superoxide anions and nitric oxide^15^. Our previous study also indicated that MIF levels in plasma are up-regulated earlier than the increase of inflammatory cytokines such as IL-6 and TNF-α in glycerol-induced AKI mice, while HSA-Trx significantly suppressed the increase of MIF as well as IL-6 and TNF-α in plasma^22^. In the present study, we also showed that the HSA-Trx inhibited an increased expression of MIF in kidney and lung (Fig. 7). These findings suggest that HSA-Trx exerts its anti-inflammatory activities, in part, from modulation of MIF levels.

As shown in Fig. 4, HSA-Trx inhibited neutrophil infiltration into lung. Previous studies reported that Trx exhibits anti-inflammatory activities via anti-chemotaxis^38^ and anti-adhesion effects^39^, resulting in a reduced number of neutrophils in the lung. Nakamura et al. reported that exogenous recombinant human Trx treatment markedly suppressed lipopolysaccharide-induced neutrophil extravasation in addition to inhibiting p38 MAPK activation in neutrophils and the shedding of L-selectin from the surface of neutrophils^38^. Hara et al. also revealed that exogenous administration of recombinant human Trx inhibited the adhesion of neutrophils on the endothelial cells^39^. Moreover, our previous study showed that HSA-Trx possessed scavenging activity of ROS released from neutrophils in vitro^19^. These findings suggested that HSA-Trx also exerted its anti-chemotactic, anti-adhesion and anti-oxidative activities against neutrophils.

Apoptosis is considered an important mode of cell death in ischemic AKI and its associated lung injury. The present study showed that HSA-Trx markedly suppresses the increased number of TUNEL-positive cells in kidney, lung and liver (Figs. 1, 5 and 6). Recently, Fujino et al. demonstrated the role of endogenous Trx on apoptosis affected by cellular redox balance^40^. Under normal conditions, apoptosis signal regulating factor 1 (ASK1) is bound to Trx. When exposed to oxidative stress, ASK1 dissociates from Trx after oxidation of Trx, and interacts with TRAF2/6, leading to apoptosis via the phosphorylation of p38 and JNK MAPK^40^. Consequently, one of the possible mechanisms that HSA-Trx inhibits apoptosis could be via the protection of intracellular Trx from ROS-induced oxidation, or the complementation of intracellular Trx, which can bind to ASK1. Moreover, the suppressive effect of HSA-Trx against increased pro-inflammatory cytokines, IL-6 and TNF-α could also be involved in the suppression of apoptosis signaling.

The present study suggests that HSA-Trx suppresses AKI-associated lung injury due to both (1) direct action, by suppression of neutrophil infiltration, inflammation and apoptosis in lung, and (2) indirect action, by ameliorating kidney injury. However, the quantitative contribution of the direct action of HSA-Trx to ameliorate lung injury in this animal model is unclear. In addition, the present study did not show the effect of HSA-Trx on hydrostatic edema, which is generally recognized as a major cause of pulmonary dysfunction following AKI due to fluid overload. To address these issues, further investigation is required using renal injury-independent conditions, such as a bilateral nephrectomy model.

To conclude, we have demonstrated that HSA-Trx may be therapeutically beneficial in preventing both cardiac surgery-associated AKI and the associated induced distant organ damage. This therapeutic action of HSA-Trx arises from its systemic and sustained multiple biological activity.

## Methods

### Expression and purification of HSA-Trx fusion protein

The *Pichia* Expression Kit was purchased from Invitrogen (Carlsbad, CA). The production and purification of HSA-Trx was performed in accordance with a previously reported method^17,22^.

### Mouse model of AKI-associated lung injury

C57BL/6 N mice (male, 8 weeks, Japan SLC, inc., Shizuoka, Japan) were maintained in a room under controlled temperature conditions with a 12 h light and 12 h dark cycle (light 8 am–8 pm) and freely provided with food and water. All animal experiments were conducted using procedures approved by the experimental animal ethics committee at Kumamoto University. To induce AKI-associated lung injury, both renal pedicles of mice were clamped for 60 min, as described in detail previously^9,25^. The mixture of HSA plus Trx or HSA-Trx alone was administered *i.v.* at a dose of 400 nmol/kg, immediately and 24 h after renal IR. An equivalent amount of phosphate-buffered saline (PBS) was administered to sham operation-treated mice and renal IR-treated mice. Details of the procedures are given in the supplemental data.

### Biochemical evaluation of blood samples

The mean blood urea nitrogen (BUN) and serum creatinine (Scr) levels were determined by a FUJI DRI-CHEM 7000 and DRI-CHEM slides system (FUJIFILM, Tokyo, Japan). The mean aspartate aminotransferase (AST) and alanine aminotransferase (ALT) levels were determined using a Transaminase C2-Test kit (Wako Pure Chemical, Osaka, Japan).

### Analysis of lung lavage samples

Analysis of bronchoalveolar lavage (BAL) samples was performed as described previously^19^. Briefly, the mice were euthanized and then BAL fluid (BALF) was collected. BALF cells were then applied to flow cytometry. The protein concentration in BALF was measured. The details are described in the supplemental data.

### Histological examination of kidney, lung and liver tissues

Harvested tissue after renal-IR were fixed in 10% formalin neutral buffer solution for 48 h and then embedded in paraffin. Kidney blocks were sectioned (2-μm), and lung and liver blocks were sectioned (4-μm). For morphological analysis, hematoxylin and eosin (HE) staining and Periodic acid-Schiff (PAS) staining were performed, and stained sections were observed using a microscope (BZ-8000; Keyence, Osaka, Japan). Four high-magnification (× 200) fields of HE-stained lung sections were randomly selected for semi-quantitative analysis of morphological alterations. All quantifications were performed using the BZ-X analyzer (Keyence) and the ImageJ software (NIH) in a blinded manner.

### Evaluation of apoptosis

For evaluation of cell apoptosis, terminal deoxynucleotidyl transferase-mediated dUTP nick-end labeling (TUNEL) staining were performed using an In situ cell death detection kit, Fluorescein (Roche, Basel, Switzerland). DAPI (Dojin Chemical, Kumamoto, Japan) was also used to detect nuclei in tissue sections. The number of TUNEL-positive cells were quantified using a BZ-X analyzer.

### Evaluation of oxidative stress and neutrophil infiltration by immunohistochemistry

Tissue sections were subjected immunohistochemistry (8-hydroxy-2-deoxyguanosine (8-OHdG), MPO and nitrotyrosine (Nitro-Tyr))^22^. The details of the procedures are described in the supplemental data.

### Measurement of lung superoxide

For evaluation of lung superoxide in situ, dihydroethidium (DHE) staining was performed^41^. A detailed description of the staining protocol is given in the supplemental data.

### Quantification of plasma pro-inflammatory cytokine levels

IL-6 and TNF-α ELISA kit were purchased from Biolegend (San Diego, CA). Plasma IL-6 and TNF-α concentrations were determined according to the manufacturer's protocol.

### mRNA expression analysis

Real-time reverse transcription-polymerase chain reaction (RT-PCR) was performed^42^. The details of the procedure are described in the supplemental data.

### Western blot analysis of MIF expression in kidney and lung tissue

For evaluation of MIF chemokine in the kidney and the lung of renal IR-treated mice, Western blotting were performed^22^. Details of the procedure are given in the supplemental data.

### Survival analysis

For the assessment of the effect of HSA-Trx on survival rate, mice were subjected to clamping both renal pedicles for 60 min. The mice were intravenously administered PBS or HSA-Trx immediately and 24 h after reperfusion. Mice were then monitored for 7 days.

### Statistical analyses

Statistical analyses were determined by analysis of variance followed by Tukey's multiple comparison. For the survival study, Kaplan–Meier survival curves and the log-rank test were used. All data are expressed as the mean ± SD. P value < 0.05 was considered statistically significant.

## Supplementary information

Supplementary Information
