# Supplementary material for: Systemic and sustained thioredoxin analogue prevents acute kidney injury and its-associated distant organ damage in renal ischemia reperfusion injury mice
Source: Sci Rep. 2020 Nov 26;10:20635. doi: 10.1038/s41598-020-75025-5 (PMC7691343; doi:10.1038/s41598-020-75025-5)
Supplement: Supplementary file 1 — Supplementary Information [file 41598_2020_75025_MOESM1_ESM.docx]

**Systemic and sustained thioredoxin analogue prevents acute kidney injury and its-associated distant organ damage in renal ischemia reperfusion injury mice**

**Kento Nishida, Hiroshi Watanabe, Masako Miyahisa, Yuto Hiramoto, Hiroto Nosaki, Rui Fujimura, Hitoshi Maeda, Masaki Otagiri, and Toru Maruyama**

**Supplemental Information**

**Supplemental Figure 1**

**Supplemental Fig. 1. Kidney and lung injury in renal IR-treated mice.**

(**A**) AKI-associated lung injury was induced by renal ischemia/reperfusion injury (IRI) where both renal pedicles were clamped for 60 min. (**B**) Changes in the levels of blood urea nitrogen (BUN) and serum creatinine (Scr) after renal IR. (**C**) Changes in protein concentration in bronchoalveolar lavage fluid (BALF) after renal IR. The mice showed elevations in BUN and Scr in a time-dependent manner until 36 hr after renal IR. Protein levels in BALF increased at 24 and 36 hr after renal IR. Data are expressed as means ±SD (n=4). *P<0.05, **P<0.01 compared with sham.

**Supplemental Figure 2**

**Supplemental Fig. 2. Plasma cytokine level and neutrophil infiltration into lung of renal IR-treated mice.**

Changes in (**A**) the levels of plasma IL-6, (**B**) the percentage of neutrophil in bronchoalveolar lavage fluid (BALF) and (**C**) the levels of plasma TNF-α after renal IR. Renal IRI-induced lung injury model mice showed an increase of plasma IL-6 and neutrophils in BALF, which reached a peak at 4 and 8 hr after renal IR, respectively. TNF-α was elevated in a time-dependent manner until 36 hr after renal IR. Data are expressed as means ±SD (n=4). *P<0.05, **P<0.01 compared with sham.**Supplemental Figure 3**

**Supplemental Fig. 3. Effect of HSA-Trx on chemokine expression in kidney and liver of renal IR-mice.**

mRNA expressions of CXCL1 and CXCL2 in (**A**) kidney and (**B**) liver of mice at 4 hr after renal IR were determined by real-time RT-PCR. Data are expressed as means ±SD (n=5–6). *P<0.05, **P<0.01 compared with sham.**Supplemental Figure 4**

**Supplemental Fig. 4. Complete western blot images before cropping into result figures.**

**Supplemental Figure 5**

**Supplemental Fig. 5. Complete western blot images before cropping into result figures.**

**Supplemental Table**

**Supplemental Table. Primers used in real-time RT-PCR.**

**Expanded Material and Methods:**

**Mouse model of acute kidney injury (AKI)-associated lung injury**

All animal experiments were performed according to the guidelines, principles, and procedures for the care and use of laboratory animals of Kumamoto University. C57BL/6N mice (male, 8 weeks, Japan SLC Inc., Shizuoka, Japan) were maintained in a temperature-controlled room with a 12 hr dark/light cycle and *ad libitum* access to food and water. To induce AKI-associated lung injury, both renal pedicles were clamped for 60 min, as described in detail previously ^1,2^. The mice were administered intravenously with phosphate-buffered saline (PBS) as control, a 1:1 mixture of human serum albumin (HSA) and thioredoxin-1 (Trx) (400 nmol/kg) or HSA-Trx fusion protein (HSA-Trx) (400 nmol/kg) immediately and 24 hr after reperfusion. After collecting blood samples for the determination of blood urea nitrogen (BUN), serum creatinine (Scr), aspartate aminotransferase (AST), alanine aminotransferase (ALT), inflammatory cytokines and chemokines, the mice were sacrificed under anesthesia at each time following reperfusion. The right kidneys were harvested and bisected in the equatorial plane, then a part of the right kidney was homogenized for quantitative real-time reverse transcription polymerase chain reaction (RT-PCR), and the left kidney was fixed in 10% formalin neutral buffer solution and prepared for routine histological examination. Similarly, the lung and liver were harvested and sectioned, then a part of the lung and liver were homogenized for quantitative RT-PCR. The remaining parts were fixed in 10% formalin neutral buffer solution and prepared for routine histological examination.

**Analysis of lung lavage samples**

Analysis of lung lavage samples was performed as described in a previous report ^3^. The mice were anesthetized and then bronchoalveolar lavage fluid (BALF) was collected by cannulating the trachea and lavaging the lung with 1 mL of sterile PBS containing 50 U/mL heparin (two times). About 1.8 mL of BALF was routinely recovered from each animal. The BALF was centrifuged at 4,100 × g for 5 min at 4°C to separate the cells in the BALF from the liquid. Cells were suspended in 0.9% NaCl and the resulting lysate was centrifuged again. BALF cells were then incubated with PerCP-Cy^TM^5.5 rat anti-mouse Ly-6G antibody (BD Biosciences, Franklin Lakes, NJ; cat#:560602) and FITC rat anti-mouse CD11b antibody (BD Biosciences, cat#:553310) in PBS at 4°C for 30 min, and washed in PBS. The cells were then applied to flow cytometry according to the instruction manual. The protein concentration in BALF was measured with the Protein Assay Coomassie Brilliant Blue solution with bovine serum albumin (BSA) as a standard.

**Evaluation of oxidative stress and neutrophil infiltration by immunohistochemistry**

Tissue sections were subjected to immunohistochemistry (8-hydroxy-2-deoxyguanosine (8-OHdG), myeloperoxidase and nitrotyrosine (Nitro-Tyr)) as described in a previous report ^4^. For the immunohistochemistry of 8-OHdG and myeloperoxidase, first, antigen retrieval was conducted by HistoVT One (Nacalai Tesque, Kyoto, Japan) at 95°C for 30 min. A solution containing 50 mM Tris/HCl + 0.1% Tween-20 (T-TB) was then used to solubilize the tissue sections, followed by blocking with Block Ace (Dainippon Pharmaceutics, Osaka, Japan) at room temperature (R/T) for 15 min. The tissue sections were reacted with the primary antibody (Goat polyclonal antibody to 8-Hydroxyguanosine; GeneTex, Irvine, CA; cat#: GTX10802 and MPO heavy chain (C-16)-R antibody, Santa Cruz Biotechnology, Santa Cruz, CA; cat#: sc-16128-R, diluted 1:50) overnight at 4°C. The tissue sections were then washed with T-TB, followed by reaction with the secondary antibody (Alexa Fluor 647 donkey anti-goat IgG (H + L) and Alexa Fluor 546 goat anti-rabbit IgG (H + L); Invitrogen, Carlsbad, CA diluted 1:200) at R/T for 1.5 hr. For the immunohistochemistry of Nitro-Tyr, first, antigen retrieval and solubilization of the tissue sections conducted, followed by treatment with 30% H_2_O_2_/methanol solutions at R/T for 30 min in order to inhibit endogenous peroxidase. The tissue sections were reacted with the primary antibody (Rabbit anti-nitrotyrosine polyclonal antibody; Chemicon International, Temecula, CA; cat#: AB5411, diluted 1:50) overnight at 4°C. The tissue sections were then reacted with Histofine Simple Stain MAX PO (R) (Nichirei Biosciences, Tokyo, Japan) at R/T for 30 min, followed by reaction with DAB solution at R/T for 3 min. After the reaction, slides were observed using a microscope (BZ-8000; Keyence, Osaka, Japan). The fluorescence from 8-OHdG were quantified using the ImageJ software (NIH). The mean fluorescence was quantified and expressed relative to values obtained in sham mice.

**Measurement of lung superoxide**

Dihydroethidium (DHE) was used to evaluate lung superoxide concentrations *in situ*, as described in detail elsewhere ^5^. After the reaction, slides were observed using a microscope (BZ-8000). DHE fluorescence of lung sections was quantified using the ImageJ software. The mean fluorescence was quantified and expressed relative to values obtained in sham mice.

**Real-time RT-PCR analysis**

Real-time RT-PCR analysis was performed as described in a previous report ^6^. In a typical run, total RNA was extracted using RNAiso PLUS (TaKaRa Bio Inc., Shiga, Japan) according to the manufacturer’s protocol. The concentration and the purity of the RNA extract were determined by the absorbance at 260 and 280 nm. The cDNA was synthesized using the PrimeScript RT master mix (TaKaRa Bio Inc.). Quantitative real-time RT-PCR analysis was performed in an iCycler thermal cycler (Bio-Rad, Hercules, CA) with an iQ5 qRT-PCR detection system attached (Bio-Rad) using SYBR Premix Ex TaqII (TaKaRa Bio Inc.). Polymerase chain reaction amplifications were performed under the following conditions: 95°C for 3 min, for 40 cycles at 95°C for 10 s (denaturation step), at 60°C for 1 min (annealing/extension steps). The sequences of the oligonucleotide primers used are shown in Supplemental Table. The threshold cycle (Ct) values for each gene amplification were normalized by subtracting the Ct value calculated for internal standard.

**Western blot analysis of macrophage migration inhibitory factor (MIF) expression in kidney and lung tissue**

Western blotting of MIF chemokine in kidney and lung tissue after renal IR was performed as described in a previous report ^4^. Each supernatant of homogenized tissue was separated by 12.5% SDS-PAGE and transferred onto polyvinylidene difluoride membranes (Immobilon-P; Millipore,Billerica, MA) by wet electroblotting. The membranes were washed three times with Tris-buffered saline containing 0.1% Tween 20 (TBS-T) and then blocked with 5% skimmed milk in TBS-T for 1 hr at R/T. The membranes were washed three times with TBS-T and then incubated overnight at 4°C with the primary antibody (MIF antibody [N1C3]; GeneTex, cat#: GTX101162, diluted 1:500 and monoclonal anti-β-actin; Sigma-Aldrich, St Louis, MO; cat#: A5411, diluted 1:5000) in TBS-T. The membranes were washed three times with TBS-T and then incubated with the secondary antibody (mouse anti-rabbit IgG-HRP; Santa Cruz Biotechnology, cat#: sc-2357, diluted 1:2000 and m-IgGκ BP-HRP; Santa Cruz Biotechnology, cat#: sc-516102, diluted 1:5000) in TBS-T for 1 hr at R/T. The membranes were washed three times with TBS-T, and immunoblots were visualized using the SuperSignal West Pico chemiluminescent substrate (Pierce Biotechnology Inc., Rockford, IL) or ImmunoStar LD (FUJIFILM, Tokyo, Japan) with LAS-4000EPUVmini (GE Healthcare, Amersham, UK). The intensity of each band was quantified using ImageJ software.

**Supplemental References**

1 Hassoun, H. T. *et al.* Ischemic acute kidney injury induces a distant organ functional and genomic response distinguishable from bilateral nephrectomy. *Am J Physiol Renal Physiol* **293**, F30-40, doi:10.1152/ajprenal.00023.2007 (2007).

2 Hassoun, H. T. *et al.* Kidney ischemia-reperfusion injury induces caspase-dependent pulmonary apoptosis. *Am J Physiol Renal Physiol* **297**, F125-137, doi:10.1152/ajprenal.90666.2008 (2009).

3 Tanaka, R. *et al.* Long-acting human serum albumin-thioredoxin fusion protein suppresses bleomycin-induced pulmonary fibrosis progression. *J Pharmacol Exp Ther* **345**, 271-283, doi:10.1124/jpet.112.201814 (2013).

4 Nishida, K. *et al.* Renoprotective effect of long acting thioredoxin by modulating oxidative stress and macrophage migration inhibitory factor against rhabdomyolysis-associated acute kidney injury. *Sci Rep* **5**, 14471, doi:10.1038/srep14471 (2015).

5 Enoki, Y. *et al.* Indoxyl sulfate potentiates skeletal muscle atrophy by inducing the oxidative stress-mediated expression of myostatin and atrogin-1. *Sci Rep* **6**, 32084, doi:10.1038/srep32084 (2016).

6 Enoki, Y. *et al.* Potential therapeutic interventions for chronic kidney disease-associated sarcopenia via indoxyl sulfate-induced mitochondrial dysfunction. *J Cachexia Sarcopenia Muscle* **8**, 735-747, doi:10.1002/jcsm.12202 (2017).
